# Locoregional therapies for the treatment of locally advanced hepatocellular carcinoma

**DOI:** 10.1590/0100-3984.2021.54.3e3

**Published:** 2021

**Authors:** Joaquim Maurício da Motta-Leal-Filho

**Affiliations:** 1 Interventional Radiologist, Department of Radiology, Instituto do Câncer do Estado de São Paulo (Icesp) and Instituto do Coração do Hospital das Clínicas da Faculdade de Medicina da Universidade de São Paulo (InCor/HC-FMUSP), São Paulo, SP, Brazil. Email: jmaufi@gmail.com.

Liver cancer is the third leading cause of cancer-related mortality worldwide. In Brazil, it is responsible for 0.7% of all tumors, with a five-year incidence of 2.7 new cases per 100,000 population^(1)^. Hepatocellular carcinoma (HCC) is the most common type, accounting for 85-90% of cases^(1)^. It is associated with low cure rates and reduced long-term survival, given that only 20-25% of patients diagnosed with HCC are candidates for curative treatment, such as surgical resection or ablation of the tumor and liver transplantation^(1)^.

At the time of diagnosis, 80% of patients with HCC already have locally or regionally advanced disease-Barcelona Clinic Liver Cancer (BCLC) stage B (intermediate) or C (advanced). Therefore, locoregional therapies using endovascular techniques (transcatheter intra-arterial therapy) play a prominent role in the treatment.

In the study conducted by Llovet et al.^(2)^, published in 2002, chemoembolization was proven to be an efficacious means of increasing survival in patients with locally advanced HCC^(2,3)^. In fact, several modalities of transcatheter intra-arterial therapy have been shown to increase survival in such patients: transarterial embolization (TAE), which involves tumor embolization without the use of chemotherapy; transarterial chemoembolization (TACE), including conventional TACE (cTACE), which involves the use of an embolic agent plus a chemotherapeutic agent (doxorubicin or cisplatin) and drug-eluting bead TACE (DEB-TACE), which involves the use of microspheres loaded with a chemotherapeutic agent; and selective internal radiation therapy (SIRT, previously known as transarterial radioembolization), which involves the use of particles labeled with the beta-emitting radioisotope yttrium-90. All of those modalities are aimed at inducing tumor necrosis, based on the fact that the vascularization of an HCC is predominantly arterial (the embolic agents occlude the vessels, resulting in ischemia), whereas most of the liver parenchyma receives its blood supply from the portal vein^(4)^. The ischemic effect can be enhanced by intra-arterial chemotherapy or radiotherapy.

For several decades, the gold standard treatment for intermediate (BCLC stage B) HCC is TACE, which can be performed using different techniques^(5)^: with particles of varying sizes, calibrated or not; with lipiodol (cTACE); or with a variety of chemotherapeutic agents (typically doxorubicin). It can be performed in a single session or over multiple sessions (two to three, on average), depending on the response of the patient^(5)^. More recently, DEB-TACE was developed in order to improve the results of TACE for HCC. To date, it has not been possible to prove that DEB-TACE is superior to TACE in terms of the results obtained, although the former has broadened the spectrum of patients who can benefit from the treatment-including patients with more severe liver dysfunction-because it minimizes side effects by lowering the peak plasma concentration of the chemotherapeutic agent^(6,7)^. It is also noteworthy that DEB-TACE has made it possible to standardize the chemoembolization technique. However, there are many clinicians who believe in the embolic effect of TAE but do not believe in the potential of combining it with chemotherapy^(8)^.

It is also possible to use TACE to rescue a patient who at first assessment does not meet the criteria for placement on a liver transplant waiting list (i.e., does not meet the Milan Criteria). That is known as downstaging, which can offer the patient the possibility of a cure. Conversely, TACE can be used to keep a patient on a liver transplant waiting list for longer (bridging), preventing the progression of the disease, in countries where the waiting list for liver transplantation is longer than six months^(9)^.

In patients with HCC classified as intermediate or advanced (BCLC stage B or C) who are not candidates for TACE or for treatment with sorafenib, the treatment most often employed is SIRT^(5)^. However, it is a costly treatment and is not widely available. Although SIRT is considered a palliative treatment, there have been reports of patients in whom HCC was downstaged after SIRT and who subsequently underwent tumor resection, liver transplantation, or both, thus being given the possibility of a cure^(10)^.

Locoregional therapies using endovascular techniques can also be combined with other locoregional therapies, such as ablation and pharmacotherapy. Interventional radiology plays a major role in the treatment of HCC. For those who wish to gain a better understanding of these therapeutic modalities, I recommend reading the article authored by Inchingolo et al.^(5)^, published in the previous issue of Radiologia Brasileira, which artfully summarizes the advantages and disadvantages of each of the methods mentioned here.
